# Dual-Task nTMS Mapping to Visualize the Cortico-Subcortical Language Network and Capture Postoperative Outcome—A Patient Series in Neurosurgery

**DOI:** 10.3389/fonc.2021.788122

**Published:** 2022-01-21

**Authors:** Ann-Katrin Ohlerth, Roelien Bastiaanse, Lyndsey Nickels, Beate Neu, Wei Zhang, Sebastian Ille, Nico Sollmann, Sandro M. Krieg

**Affiliations:** ^1^ Center for Language and Cognition Groningen, Groningen, Netherlands; ^2^ International Doctorate in Experimental Approaches to Language and Brain (IDEALAB, Universities of Groningen, Potsdam, Newcastle, and Macquarie University), Sydney, NSW, Australia; ^3^ Center for Language and Brain, Higher School of Economics, National Research University, Moscow, Russia; ^4^ School of Psychological Sciences, Faculty of Medicine, Health and Human Sciences, Macquarie University, Sydney, NSW, Australia; ^5^ Department of Neurosurgery, School of Medicine, Klinikum rechts der Isar, Technical University of Munich, Munich, Germany; ^6^ Department of Diagnostic and Interventional Radiology, University Hospital Ulm, Ulm, Germany; ^7^ Department of Diagnostic and Interventional Neuroradiology, School of Medicine, Klinikum rechts der Isar, Technical University of Munich, Munich, Germany; ^8^ TUM-Neuroimaging Center, Klinikum rechts der Isar, Technical University of Munich, Munich, Germany

**Keywords:** language mapping, postoperative language state, navigated transcranial magnetic stimulation, action naming, object naming, direct electrical stimulation, case series

## Abstract

**Background:**

Perioperative assessment of language function in brain tumor patients commonly relies on administration of object naming during stimulation mapping. Ample research, however, points to the benefit of adding verb tasks to the testing paradigm in order to delineate and preserve postoperative language function more comprehensively. This research uses a case series approach to explore the feasibility and added value of a dual-task protocol that includes both a noun task (object naming) and a verb task (action naming) in perioperative delineation of language functions.

**Materials and Methods:**

Seven neurosurgical cases underwent perioperative language assessment with both object and action naming. This entailed preoperative baseline testing, preoperative stimulation mapping with navigated Transcranial Magnetic Stimulation (nTMS) with subsequent white matter visualization, intraoperative mapping with Direct Electrical Stimulation (DES) in 4 cases, and postoperative imaging and examination of language change.

**Results:**

We observed a divergent pattern of language organization and decline between cases who showed lesions close to the delineated language network and hence underwent DES mapping, and those that did not. The latter displayed no new impairment postoperatively consistent with an unharmed network for the neural circuits of both object and action naming. For the cases who underwent DES, on the other hand, a higher sensitivity was found for action naming over object naming. Firstly, action naming preferentially predicted the overall language state compared to aphasia batteries. Secondly, it more accurately predicted intraoperative positive language areas as revealed by DES. Thirdly, double dissociations between postoperatively unimpaired object naming and impaired action naming and vice versa indicate segregated skills and neural representation for noun versus verb processing, especially in the ventral stream. Overlaying postoperative imaging with object and action naming networks revealed that dual-task nTMS mapping can explain the drop in performance in those cases where the network appeared in proximity to the resection cavity.

**Conclusion:**

Using a dual-task protocol for visualization of cortical and subcortical language areas through nTMS mapping proved to be able to capture network-to-deficit relations in our case series. Ultimately, adding action naming to clinical nTMS and DES mapping may help prevent postoperative deficits of this seemingly segregated skill.

## Introduction

Preoperative assessment of language and delineation of functional tissue in relation to a lesion is crucial to perform optimal neurosurgical intervention, while aiming to prevent post-operative deficits in brain tumor patients (1, 2). In addition to imaging tools such as functional magnetic resonance imaging (fMRI), preoperative language mapping with navigated transcranial magnetic stimulation (nTMS) has recently gained increasing interest. This non-invasive stimulation tool allows preoperative mapping of cortical functions by inducing transient lesions in small anatomical areas (3). This enables the mapping of areas associated with language disruption, which can be used in guiding surgical planning and intraoperative tumor resection (4–6). As the closest analogue to the gold standard of intraoperative mapping with direct electrical stimulation (DES), nTMS mapping as well relies on error elicitation during language production, and has shown superiority to fMRI by demonstrating a better overlap of nTMS results with DES results (4, 7–11).

The full potential of nTMS is reached in combination with fiber tracking, where language-positive nTMS spots can be employed for visualization of functionally involved subcortical white matter tracts (12–14). Using this preoperative visualization of the language network in relation to the lesion has proven its usefulness in neurosurgical practice by enlarging the extent of resection and the confidence of the surgeon, while leading to fewer language deficits at discharge (6, 13, 15).

Even though nTMS is generally established as a validated method that has been incorporated well in the clinical workflow in an increasing number of neuro-oncological centers (6, 15), one area that deserves further attention is the linguistic depth of the task commonly used during nTMS mapping and its influence on successfully preventing language deficits after surgery, given a task’s crucial role for the resulting map of language-positive nTMS points. By solely employing object naming (ON), that targets noun production, for both cortical and subcortical delineation of function, more complex language skills may be neglected and endangered at the postoperative state. Consequently, it has been proposed that the addition of a simple second task of action naming (AN) may be beneficial (16, 17): By triggering the production of verbs in the context of a short sentence, grammatical and semantic skills can be tested that are not targeted by object naming and that, more importantly, are known to rely on anatomically distinct circuits (18–21). In DES mapping and in healthy participants using nTMS, this double dissociation, and the added value of action naming, have been shown to be especially important for the visualization of the semantically relevant ventral stream tracts (22–26). What is still missing, however, is the exploration of the use of both object and action naming tasks in nTMS prior to surgical procedures in tumor patients and the usefulness of this protocol for preventing postoperative language deficits. It is possible that better visualization of cortical and subcortical substrates of language functions may be achieved through addition of a second task, involving verbs.

In this case series, we deliver a description of our first experience with the dual-task mapping protocol in the clinical workflow of pre- (, intra-) and postoperative language testing considering nTMS mapping. As a first exploratory step, on the basis of patient cases harboring language-eloquent brain tumors, we evaluate the contribution of each task in (1) delineating the language state and network preoperatively, (2) informing and “predicting” the intraoperative mapping with DES through the nTMS maps, and (3) capturing the language state and network in relation to the acute postoperative outcome. We present seven cases, of which three were operated on the basis of only nTMS presurgical data and four with additional intraoperative mapping by DES in order to explore the issue of which patients may benefit from the administration of the dual-task protocol.

## Methods

### Patient Population

Between March 2021 and August 2021, fourteen patients were included in the dual-task mapping protocol. Out of these, seven showed no proximity of the lesion to the language network as mapped out by nTMS (e.g., pre-frontal lesion) and are not included in this paper.

Inclusion criteria were that patients were right-handed and had an intracranial malignant tumor in the left hemisphere in the proximity of the language network as determined by nTMS mapping. Demographics, lesion characteristics and background language assessments of the seven cases are summarized in **Table 1**.

**Table 1:** Demographics of the patient cases.

Case	Age	Gender	Lesion site	Pathology	WHO grade	Awake surgery	Preoperative language status (ACL/AAT)
1	63	M	SFG	oligodendro-glioma	III	no	moderate impairment of auditory comprehension, and reading, mild semantic fluency impairment
2	70	M	prG	glioblastoma	III	no	not administered, no impairment in spontaneous speech
3	47	F	parietal white matter	metastasis of cervix carcinoma	–	no	not administered, no impairment in spontaneous speech
4	53	M	posterior STG	glioblastoma	III	yes	moderate impairment of production, mild writing impairment, mild impairment of phonological and semantic fluency
5	38	M	SFG	anaplastic astrocytoma	III	yes	no impairment, very mild semantic fluency impairment
6	57	M	middle ITG	astrocytoma	II	yes	mild impairment of comprehension and moderate impairment in phonological fluency
7	33	M	insula	astrocytoma	II	yes	no impairment

### Preoperative MRI

Patients underwent preoperative MRI using a 3-Tesla MRI scanner (Achieva dStream or Ingenia, Philips Healthcare, Best, The Netherlands) with a 32-channel head coil. Among other sequences in the standard protocol for neuro-oncological imaging, acquisition included a three-dimensional (3D) fluid attenuated inversion recovery (FLAIR) sequence (TR/TE: 4800/277 ms, 1 mm^3^ isovoxel covering the whole head), a 3D T1-weighted turbo field echo sequence (TR/TE: 9/4 ms, 1 mm^3^ isovoxel covering the whole head) and/or 3D T1-weighted turbo spin echo black-blood sequence (TR/TE: 4000/35 ms, 1 mm^3^ isovoxel covering the whole head) without and with administration of a contrast agent (Dotagraf, Jenapharm, Jena, Germany), and a diffusion tensor imaging (DTI) sequence (TR/TE 5000/78 ms, voxel size of 2 × 2 × 2 mm^3^, 32 diffusion directions, one volume at b = 0 s/mm^2^, 32 volumes at b = 1000 s/mm^2^).

### Preoperative Language Assessment with nTMS Mapping

In order to describe the overall language status of the patient, standardized language batteries [either Aachener Aphasie Test (AAT) or Aphasie Check List (ACL)] were carried out in most patients to identify pre-existing language impairments, when deemed appropriate by the medical team.

Preoperative language mapping was conducted using the Nexstim eXimia NBS system (version 5.1; Nexstim Plc, Helsinki, Finland) and following the protocol described by Krieg et al. (27). In addition to the standard ON task implemented in the system (e.g., the patient sees a black-and-white drawing of an object and has to name it: *apple*), AN in sentence context from the VAN-POP battery (16) was administered in a second block to trigger verb production (e.g., the patient sees a black-and-white drawing of a daily activity and has to name it: *the man reads*).

For both task blocks, the following steps were taken: firstly, on the day of nTMS mapping, the patient had to name all stimuli (80 ON items and 75 AN items) without stimulation in two baseline rounds to discard all items not fluently named (all items that were named with very late onset, could not be named at all or only on the second attempt were considered as errors). The score of correctly named items during the first baseline round comprised the preoperative baseline performance for that patient on ON and AN tasks.

Secondly, nTMS language mapping covering the majority of the left hemisphere cortical surface was performed using the patient-tailored stimuli list under a 5Hz/5 pulse protocol at 110% of the previously established resting motor threshold, as described previously for ON and for AN (24, 27). Stimuli were presented with a display time of 700 ms (ON) and 1000 ms (AN), as standardized in Ohlerth et al. (16), with an inter-picture-interval of 2500ms and a picture-trigger-interval of 0 ms. The ON block was always administered first.

Baseline and stimulation naming were video-recorded and afterwards examined for naming errors by a trained neuro-linguist (AKO) (24). All stimulation sequences leading to an error were counted (**Table 2**), marked as language-positive sites, and exported *via* DICOM format to the neuro-navigation planning unit (Brainlab Elements Net server version 3.0.6.14., Brainlab AG), resulting in two separate cortical language maps, one for ON and one for AN.

**Table 2:** Proportion of induced errors during nTMS mapping of the left hemisphere with object naming (ON) and action naming (AN).

Case	Errors under nTMS in ON	Errors under nTMS in AN	Difference per Chi-square
1	0.15	0.09	0.13
2	0.15	0.15	0.87
3	0.11	0.09	0.54
4	0.04	0.09	0.05*
5	0.12	0.14	0.73
6	0.06	0.15	0.01*
7	0.16	0.25	0.07

Significant difference is marked by use of asterisk.

### Fiber Tracking on the Basis of nTMS

Deterministic fiber tracking was performed on basis of a DTI sequence with 32 diffusion directions solely using the nTMS language-positive cortical sites as regions of interest (ROIs). For ROI creation, 5 mm rims were added to each language positive cortical site (12). Based on these ROIs, fiber tracking then visualized all fibers with a fiber length of 100 mm at a predetermined fractional anisotropy value of 0.15 and an angulation of 20° (12). This resulted in two separate language-related white matter network visualizations, one for ON and one for AN, that were visually inspected for presence of the known language tracts [Arcuate Fascicle (AF), Superior Longitudinal Fascicle (SLF), Frontal Aslant Tract (FAT), Uncinate Fascicle (UF), Inferior Longitudinal Fascicle (ILF), Inferior Fronto-Occipital Fascicle (IFOF)], the speech-articulatory tract [Cortico-nuclear tract, (CNT)], and commissural fibers (CF) and their proximity to the lesion (12, 26).

### Intraoperative Mapping With DES

In order to establish an item list to be used during intraoperative mapping, for each patient, 91 new items for ON (those used in the hospital’s standard neurosurgical procedure) and the same 75 items for AN from the VAN-POP were tested again one day prior to the surgery. Any errors were noted and those items were removed from the intraoperative stimulation trials.

During surgery, first an adequate level of anesthesia and sedation was administered to ensure the patient was unconscious while the head was placed in a Mayfield head clamp, and the cortex was exposed. Then neuro-navigation was used to localize and align the cortical and subcortical anatomy in relation to the preoperative scans and mapping results. Prior to language mapping, (general) analgesia and sedation were reduced to slowly awaken the patient. Once the patient was fully awake, calm, and cooperative, language mapping commenced using a bipolar stimulation electrode (distance 5mm) with a 4s stimulation output at an intensity of 4mA and a frequency of 50Hz. The entire exposed cortex was interrogated in 5-10mm steps and each stimulated site was tested at least 3 times. First, mapping was conducted with ON only, followed by a separate mapping with AN only. Cortical sites in which stimulation resulted in at least 2 errors from 3 stimulations were considered positive for the respective task, marked by number (1-digit = ON, 2-digit = AN) and their location was transferred to the navigation system. Resection was carried out sparing all positive areas (4, 28, 29).

### Postoperative MRI and Image Co-Registration

Acquisition of MRI according to a standardized protocol in neuro-oncological patients was administered following the same sequences with the same parameters as in the preoperative stage within 48 hours of surgery. In order to assess the proximity of the resection cavity to the language networks for each task, the fiber tracking results were registered to the postoperative T1-weighted MRI datasets. Distortion correction and elastic fusion calculations were performed to account for brain shift (30). Pathological lesions, such as residual tumor or ischemia in the scans, the overlap of the cavity and the visualized language-positive cortical sites of nTMS, and/or the visualized white matter network were assessed. No case showed ischemic lesions.

### Postoperative Language Assessment

On the third postoperative day, the language status was assessed by administering ON and AN without stimulation. Presentation of items followed the same procedure as the preoperative baseline naming. As for the preoperative assessment, all items that were not named correctly and fluently were scored as errors.

### Analysis of Language State

Scores of each patient on the preoperative baseline naming and postoperative naming in ON and AN were compared to the performance of healthy control subjects [for ON performance, see (31), for AN performance, see (24)]. Results were analyzed using statistics designed for single case methodology (32) (**Table 3** and **Supplementary Material Table S1**). The change in pre- versus postoperative scores was evaluated using McNemar’s/Fisher’s exact tests. To assess the difference between ON and AN in the change in baseline versus postoperative accuracy, Wilcoxon’ two-sample tests were applied to the difference in accuracy between baseline and postoperative test results for each test. Furthermore, Spearman correlations were used to examine the relationship between error patterns and several linguistic variables of the target words [ON: length, frequency, age of acquisition, animacy, and compound status; AN: length, frequency, age of acquisition, regularity, transitivity, instrumentality, name relatedness of the target verb to a noun (16)]. Bonferroni corrections were applied to correct for multiple comparisons in these correlations (**Supplementary Material Table S2**).

**Table 3:** Proportion of correctly and fluently named items in ON and AN across testing points.

Case	Test	Baseline	1 Day Preoperative	3 Days Postoperative	Difference between Baseline and Preoperative	Difference between Baseline and Postoperative	Difference in Decline between ON and AN (unpaired MWU)
1	ON	0.813*	N/A	0.838*	N/A	0.790	0.967
	AN	0.573*#	N/A	0.600*#	N/A	0.831	
2	ON	0.850	N/A	0.825	N/A	0.803	0.702
	AN	0.747	N/A	0.693#	N/A	0.522	
3	ON	0.975	N/A	0.988	N/A	0.999	0.567
	AN	0.880	N/A	0.867	N/A	0.999	
4	ON	0.925	0.868*	0.747*	0.147	0.037*	0.005*
	AN	0.773	0.653*#	0.427*#	0.039*	<0.001*	
5	ON	0.963*	0.934*	0.888	0.505	0.114	0.555
	AN	0.733*	0.680*#	0.707#	0.480	0.831	
6	ON	0.888	0.945*	0.350*#	0.263	<0.001*	0.004*
	AN	0.813	0.733*	0.533*#	0.286	<0.001*	
7	ON	0.913	0.967*	0.875*	0.192	0.606	0.040*
	AN	0.787	0.827*	0.587*#	0.580	0.009*	

^1^Scores that indicate a significant impairment (p<.05) compared to healthy normative data (Singlims, Crawford et al., 2010) are marked by a hashtag (#). Healthy controls performed at 0.884 ± 0.046 (range 0.8-0.933) for ON (31) and at 0.852 ± 0.059 (range 0.667-0.933) for AN (26). Performance where the patients show a significantly bigger difference between tasks compared to controls (Revised Standardized Difference Test (RSDT), Crawford et al., 2010) are marked by an asterisk (*). All p values are 2-tailed. Cases 1-3 were not operated awake and, hence, the tests were not administered (N/A) 1 Day preoperatively.

## Case Descriptions

### Clinical Case 1

This patient was a 63-year-old male, admitted to hospital after a general epileptic seizure. Scans showed a lesion in the superior frontal gyrus (SFG). During preoperative language assessment, the patient scored poorly on reading and displayed a moderate auditory comprehension impairment and mild semantic fluency deficiency. Baseline naming with ON and AN showed that AN was clinically impaired compared to healthy control participants as well as significantly worse than ON (**Table 3** and **Supplementary Material Table S1** for all scores and comparisons).

**Figure 1:**
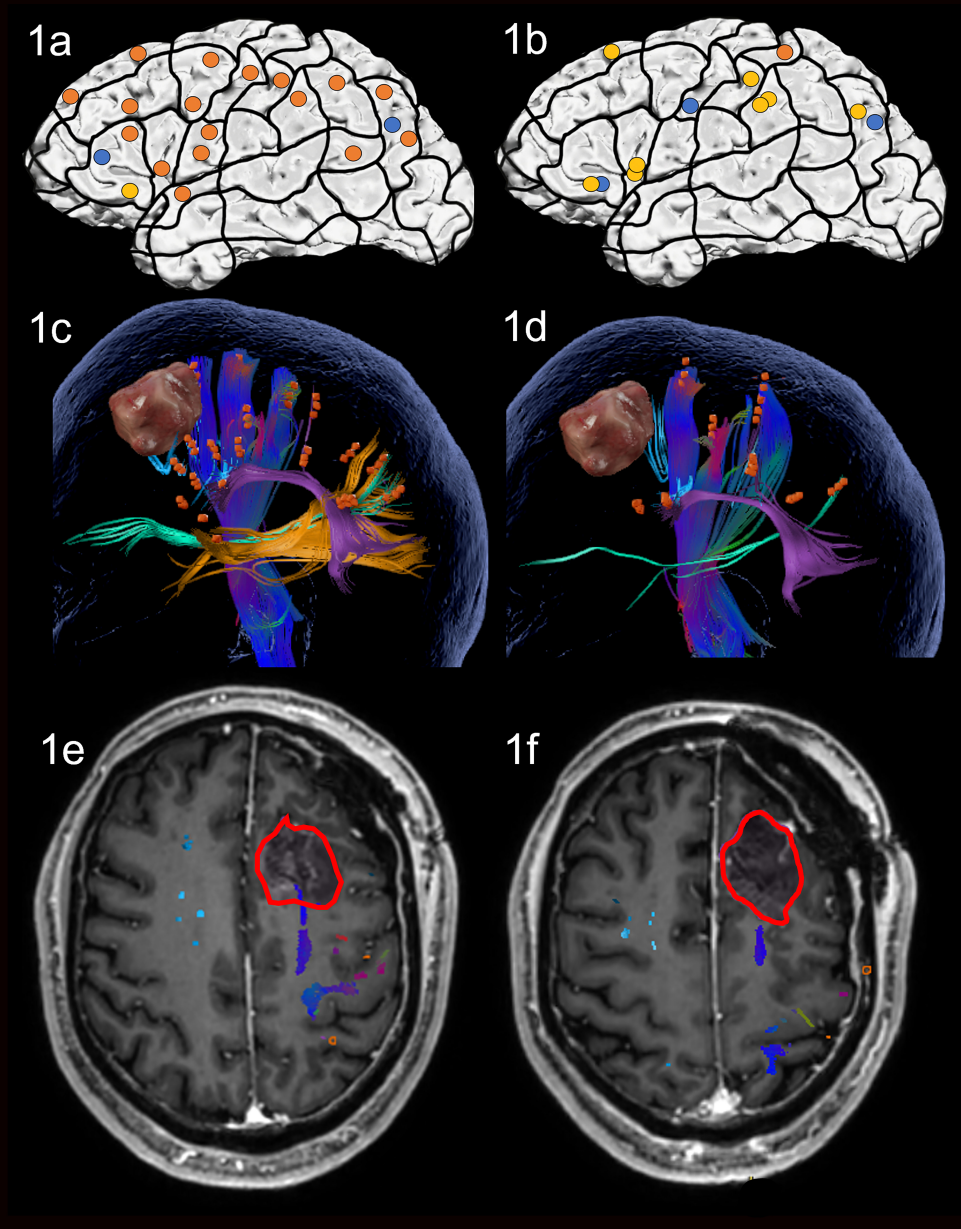
Case 1. Left panels show ON results, right panels AN results. Panels **(A, B)** nTMS stimulation sites that induced errors; no response and hesitations in orange, semantic errors in yellow and performance errors in blue. Panels **(C, D)** Fiber tracking results including the lesion, nTMS cortical endpoints in orange and color-coded tracts with CNT in direction-coded, here dark blue, AF in purple, IFOF in turquoise, ILF in yellow, and CF in light blue. Panels **(E, F)** Overlap of tracking and postoperative T1-weighted imaging after elastic fusion with the resection cavity marked in red.

Preoperative nTMS mapping at baseline revealed language-positive nTMS spots for ON mostly in the frontal and the parietal lobe with one spot in proximity to the lesion (**Figure 1A**). Subsequent fiber tracking for ON included the AF, ILF, IFOF, CF and CNT (**Figure 1C**). Language-positive points for AN were more scarce and overlapped nearly entirely with ON points, resulting in a similar white matter visualization of the AF, IFOF, CF and CNT (**Figures 1B, D**).

Due to the prefrontal location of the tumor, which was only in proximity to the CNT of the delineated networks, the operation was performed asleep and without complications. Gross total resection was achieved.

On the third postoperative day, the patient showed a slight but not significant improvement in both ON and AN compared to preoperative testing, however, he was still impaired, and AN continued to be significantly worse than ON. Postoperative MRI confirmed that no language tract was in the vicinity of the resection cavity but only the articulatory speech tract CNT (**Figures 1E, F**). Case 1 was discharged on the sixth postoperative day. No additional cognitive nor physical impairments were noted.

### Clinical Case 2

Case 2 was a 70-year-old male, who presented having recently experienced the loss of fine motor skills in his right foot and hand. Imaging confirmed a lesion in the precentral gyrus (prG). Apart from these issues, his cognitive skills seemed intact at the preoperative stage, supported by normal scores for baseline naming of ON and AN (**Table 3** and **Supplementary Material Table S1**).

**Figure 2:**
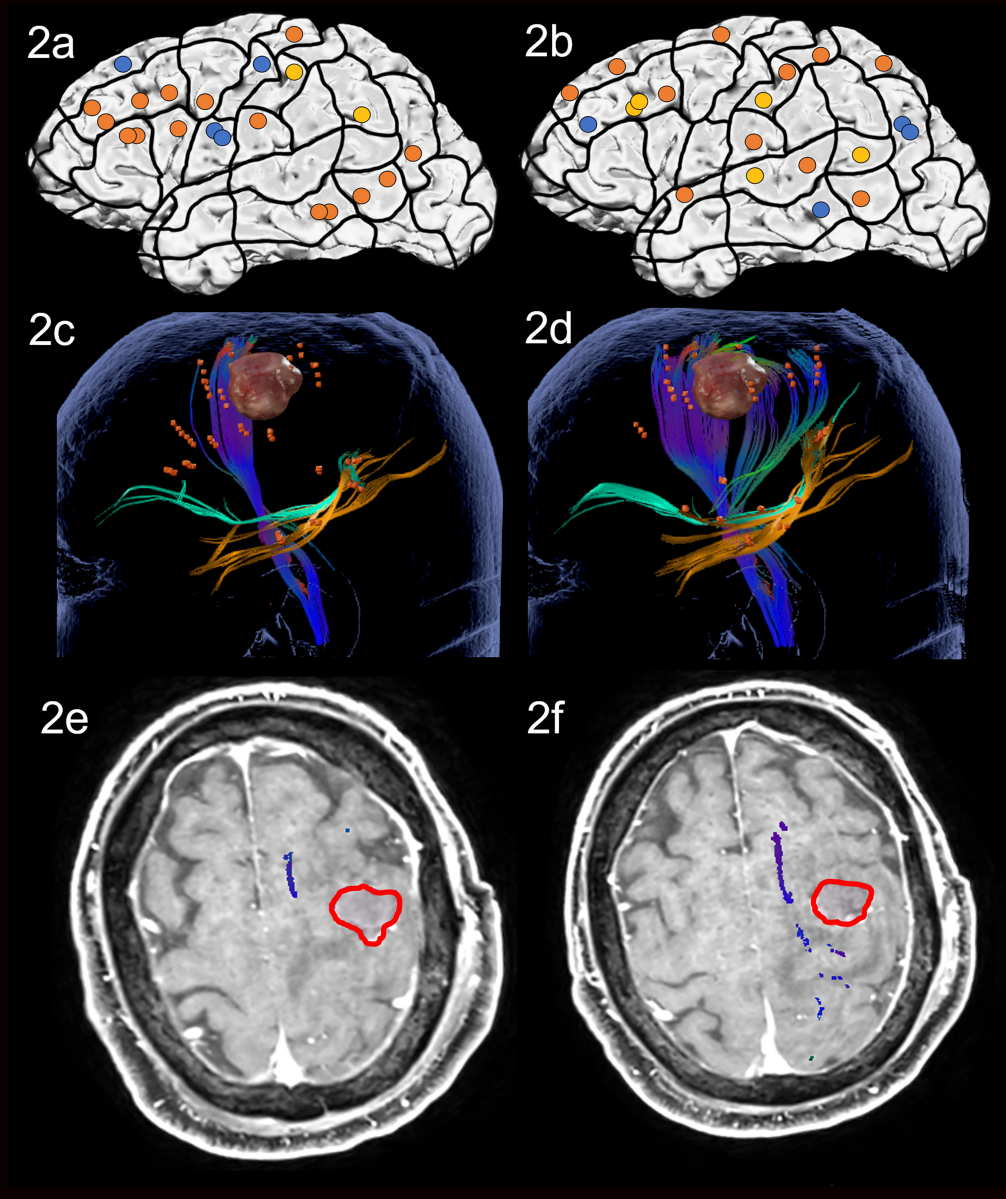
Case 2. Left panels show ON results, right panels AN results. Panels **(A, B)** nTMS stimulation sites that induced errors; no response and hesitations in orange, semantic errors in yellow and performance errors in blue. Panels **(C, D)** Fiber tracking results including the lesion, nTMS cortical endpoints in orange and color-coded tracts with CNT in direction-coded, here dark blue, IFOF in turquoise, and ILF in yellow. Panels **(E, F)** Overlap of tracking and postoperative T1-weighted imaging after elastic fusion with the resection cavity marked in red.

Preoperative nTMS mapping at baseline showed language-positive cortical areas for ON in the middle frontal regions, and in the middle temporal gyrus (MTG). There were few parietal points and 3 points in the prG itself, resulting in the visualized white matter of the CNT, ILF and IFOF (**Figures 2A, C**). For AN, nTMS mapping revealed language-positive spots in the middle and superior frontal gyrus, the superior and middle temporal gyrus, some parietal points and the poG, as well leading to visualization of the CNT, ILF and IFOF (**Figures 2B, D**). Despite language-positive points on the pre- and post central gyrus, the white matter networks of both tasks did not appear to be in proximity of the lesion. The surgery was therefore performed asleep and with no complications.

Postoperative assessment on the third postoperative day showed no significant change in the patient’s performance, in agreement with results of postoperative MRI indicating no close distance between cavity and both the networks for ON and AN (**Figures 2E, F**).

### Clinical Case 3

This patient was a 47-year-old female. She was admitted to the hospital after suffering from a focal seizure leaving her with temporary hemi-hypoesthesia and anomic aphasia, that resolved after 2 days. Scans were indicative of multiple metastases in both hemispheres, most likely secondary to a cervical carcinoma resected in 2008. Apart from three small lesions, the lesion to be resected at this point was situated in the left postcentral white matter. No cognitive or language functions seemed impaired preoperatively and she did not perform significantly differently to healthy controls for either ON or AN (**Table 3** and **Supplementary Material Table S1**).

**Figure 3:**
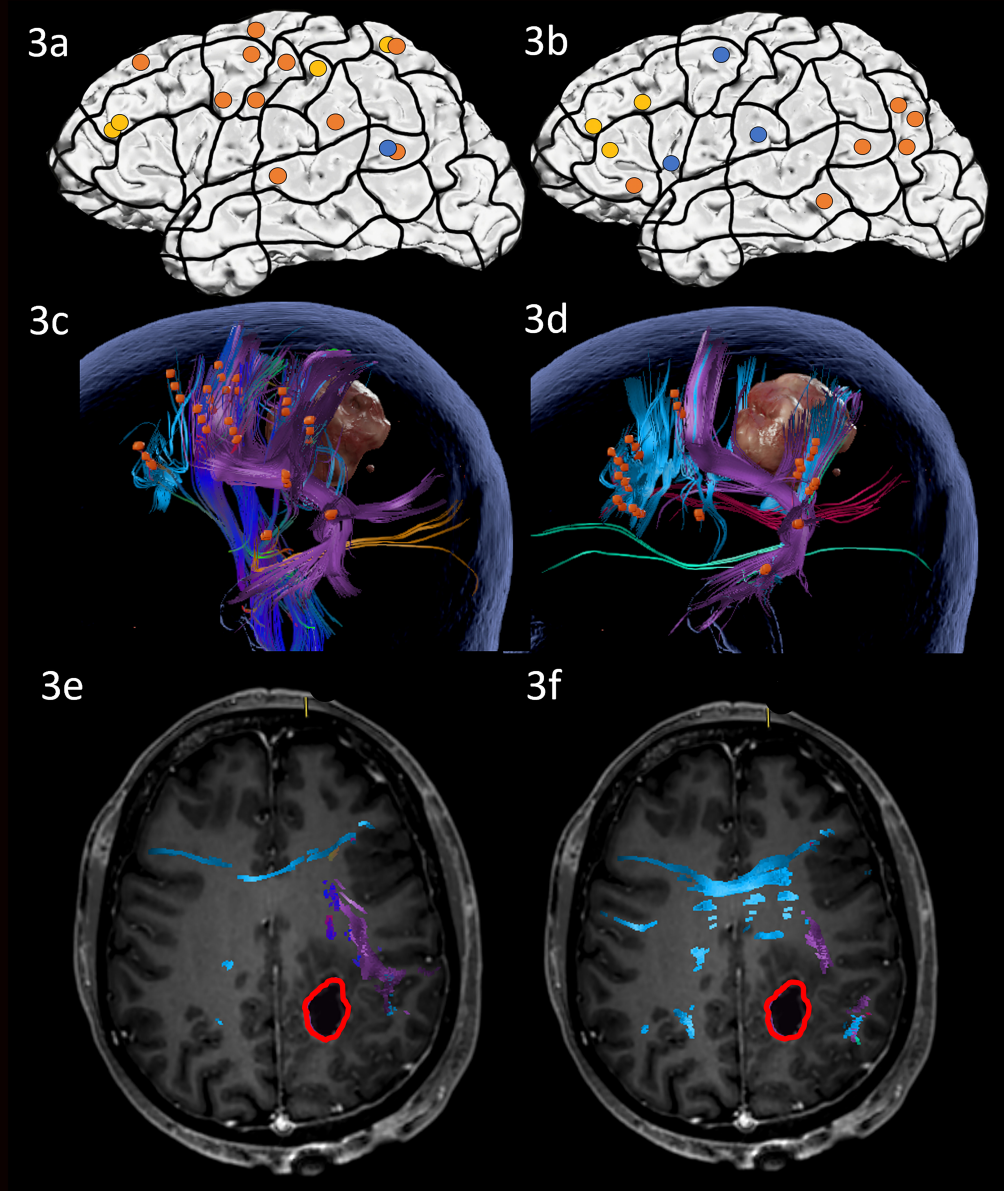
Case 3. Left panels show ON results, right panels AN results. Panels **(A, B)** nTMS stimulation sites that induced errors; no response and hesitations in orange, semantic errors in yellow and performance errors in blue. Panels **(C, D)** Fiber tracking results including the lesion, nTMS cortical endpoints in orange and color-coded tracts with CNT in direction-coded, here dark blue, AF in purple, SLF in pink, IFOF in turquoise, ILF in yellow, and CF in light blue. Panels **(E, F)** Overlap of tracking and postoperative T1-weighted imaging after elastic with the resection cavity marked in red.

Preoperative nTMS mapping at baseline revealed language-positive spots for ON in the superior frontal regions, as well as some parietal and superior temporal areas, resulting in visualization of a white matter network comprising the AF, ILF, CNT and CF (**Figures 3A, C**). Preoperative nTMS mapping with AN showed language-positive spots in more classical language areas such as the inferior frontal, and the posterior temporal and angular regions. These led to white matter visualization of the AF, SLF, IFOF and CF (**Figures 3B, D**). The surgery was performed asleep with no complications.

On the third postoperative day, the patient performed almost identically compared to the preoperative state in both ON and AN. MRI scanning demonstrated that the resection cavity did not overlap or approach the fiber tracts implicated in either ON nor AN (**Figures 3E, F**). The patient was discharged at the fifth postoperative day with no new impairments.

### Clinical Case 4

Case 4 was a 53-year-old male who was referred following an episode of aphasia in the summer of 2020 and a second in February 2021. Scans in March 2021 revealed a lesion in the posterior superior temporal gyrus (STG), confirmed as a glioblastoma at biopsy. During preoperative assessment, he showed a mild to moderate impairment in language production, a mild impairment in writing as well as low scores in phonological and semantic fluency. He exhibited no significant impairment in accuracy on either ON or AN compared to healthy controls at this point. However, for two items in AN, he displayed a tendency to paraphrase verbs (*the man does ironing* instead of *the man irons*), a pattern that is not observed in healthy controls. This worsened over the course of the month between biopsy and resection surgery: At preoperative testing one month later and one day prior to surgery, he was significantly worse, now clinically impaired on AN and displayed a tendency to paraphrase verbs in 5 items. ON remained unimpaired (**Table 3** and **Supplementary Material Table S1**).

**Figure 4:**
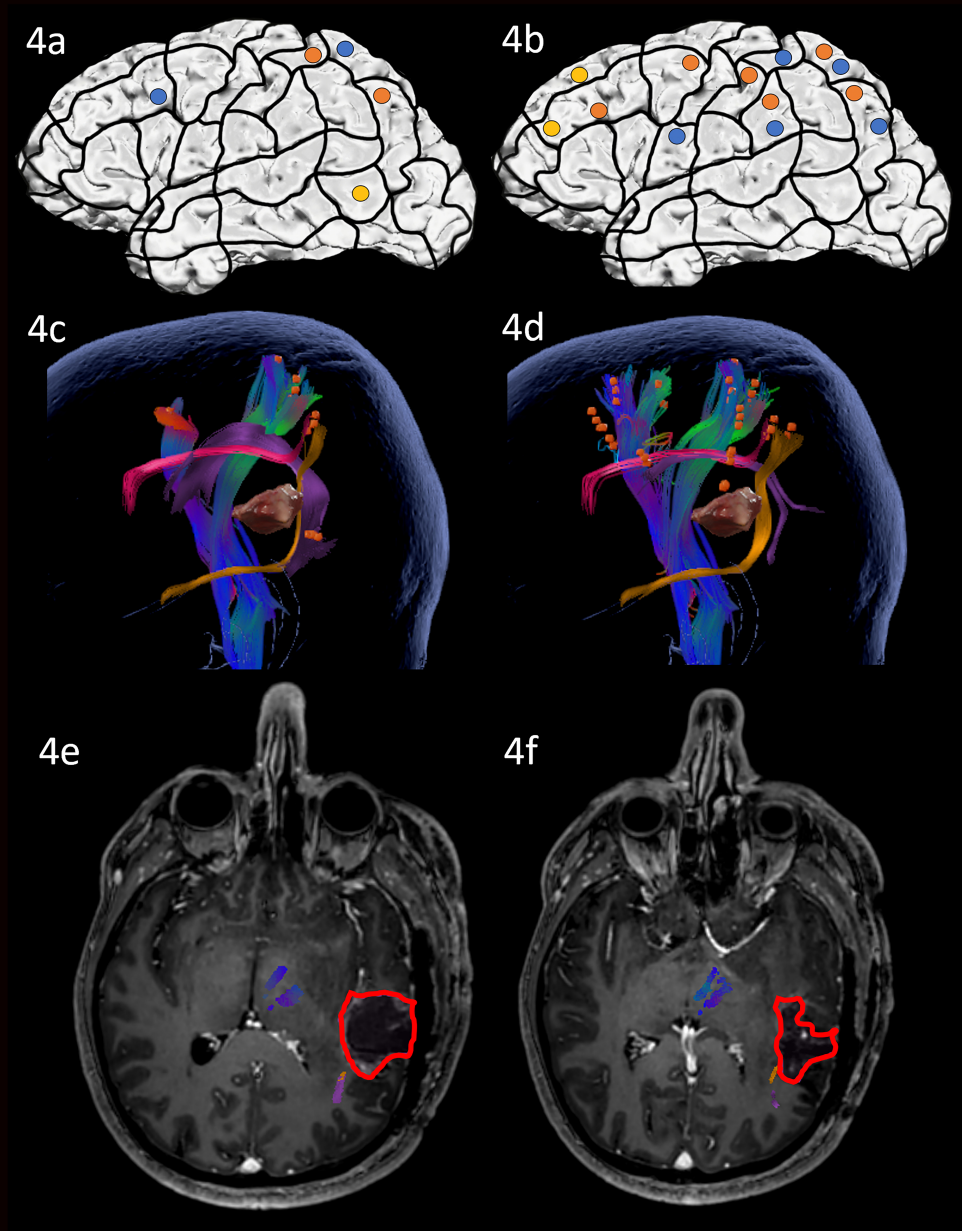
Case 4. Left panels show ON results, right panels AN results. Panels **(A, B)** nTMS stimulation sites that induced errors; no response and hesitations in orange, semantic errors in yellow and performance errors in blue. Panels **(C, D)** Fiber tracking results including the lesion, nTMS cortical endpoints in orange and color-coded tracts with CNT in direction-coded, here dark blue, AF in purple, SLF in pink, and ILF in yellow. Panels **(E, F)** Overlap of tracking and postoperative T1-weighted imaging after elastic fusion with the resection cavity marked in red.

Preoperative nTMS mapping at baseline with ON revealed only a few parietal, middle frontal and one temporal cortical language-positive spots, leading to white matter visualization of the AF, SLF, ILF and CNT (**Figures 4A, C**). During AN under nTMS mapping, middle frontal and several parietal cortical regions were uncovered, resulting in a similar white matter visualization of the AF, SLF, ILF and CNT (**Figures 4B, D**).

Due to the close location of the lesion to the language networks, an awake procedure including cortical DES mapping using both ON and AN tasks was performed. The craniotomy exposed inferior parietal and posterior STG regions. Stimulation of the exposed cortex led to only occasional errors with no site being repeatedly positive (2 out of 3 rule) in either of the tasks. Resection of the posterior STG and subsequently the underlying white matter was, therefore, carried out. There were no complications and intra-operative MRI confirmed a gross total resection.

At postoperative assessment on the third postoperative day, the patient performed significantly more poorly on both ON and even poorer on AN compared to the preoperative state. AN seemed particularly affected and had declined significantly more severely than ON. The patient now paraphrased 14 verb items that were not troublesome at baseline. Moreover, poorer accuracy in AN correlated with transitivity, meaning that more complex verbs (*the man feeds the horse* vs. *the man sleeps*) were more problematic (**Supplementary Material Table S2**). Postoperative MRI scanning confirmed that the resection cavity left most of the white matter network unaffected, but was in proximity of the ILF, especially for AN (**Figures 4E, F**). This suggests that the vulnerable ventral stream may be responsible for the worsening in AN.

The patient was discharged on the third postoperative day with no further cognitive nor physical signs of impairment except for the reported language deficit.

### Clinical Case 5

Case 5 was a 38-year-old male, who had suffered from severe headaches for a month with a subsequent general epileptic seizure. Scans revealed a large lesion in the superior frontal region. He scored within normal limits on the preoperative language batteries and on both ON and AN at the baseline, although AN was significantly less accurate than ON. A few days later, at preoperative testing one day prior to surgery, his performance was not significantly different, but now displaying impaired AN scores (**Table 3** *;* **Supplementary Material Table S1**).

**Figure 5:**
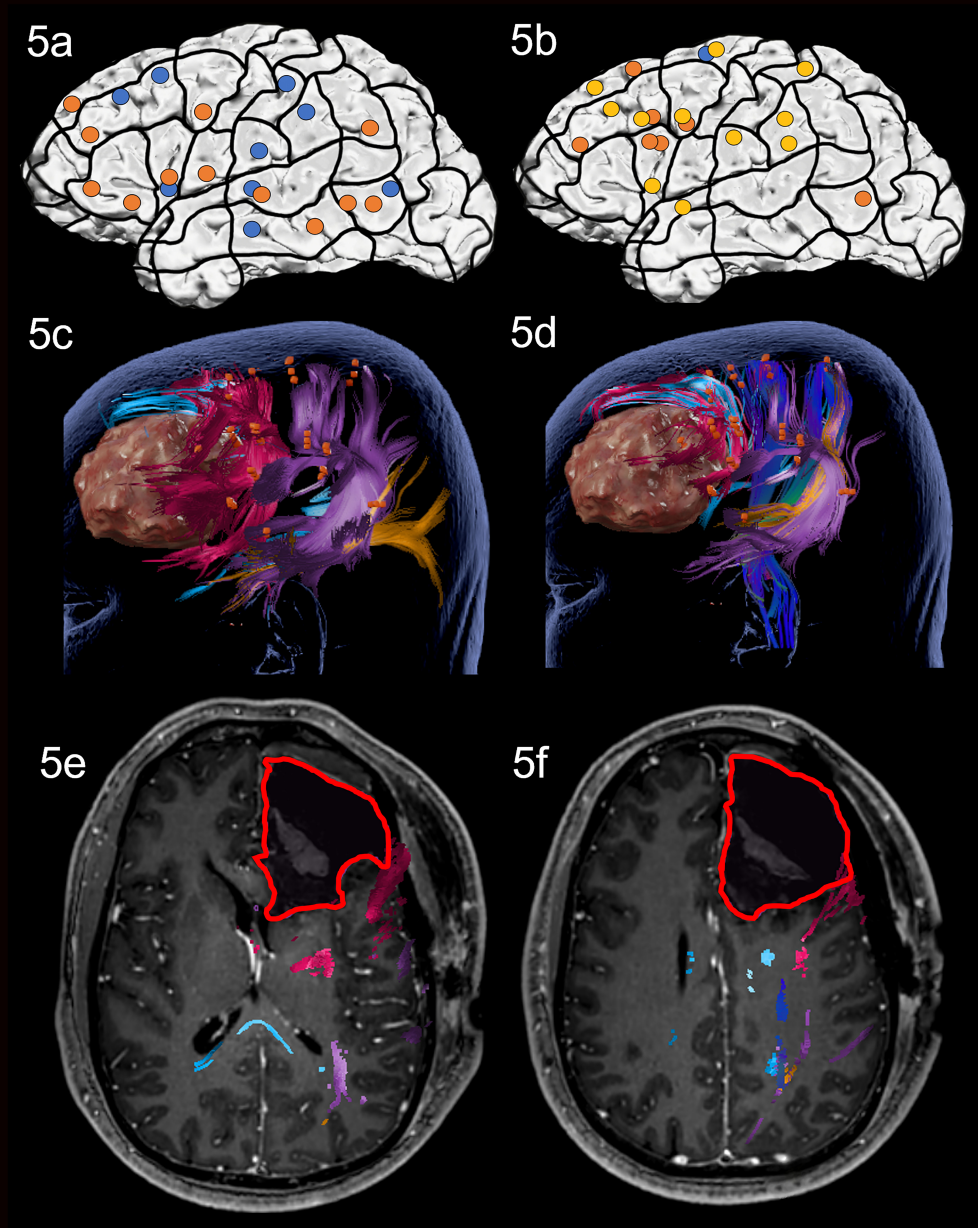
Case 5. Left panels show ON results, right panels AN results. Panels **(A, B)** nTMS stimulation sites that induced errors; no response and hesitations in orange, semantic errors in yellow and performance errors in blue. Panels **(C, D)** Fiber tracking results including the lesion, nTMS cortical endpoints in orange and color-coded tracts with CNT in direction-coded, here dark blue, AF in purple, ILF in yellow, Frontal Aslant Tract in pink, and CF in light blue. Panels **(E, F)** Overlap of tracking and postoperative T1-weighted imaging after elastic fusion with the resection cavity marked in red.

During nTMS mapping at baseline, a large network was revealed for ON covering all three lobes and leading to a white matter visualization of the AF, ILF, CF and the Frontal Aslant Tract (**Figures 5A, C**). The nTMS mapping with AN led to many semantic errors and showed a similarly distributed network to ON with a high number of frontal areas and a white matter visualization of the AF, ILF, CNT, CF and Frontal Aslant Tract (**Figures 5B, D**).

Due to the close proximity to known frontal language areas and the visualized networks, the operation was performed awake with intraoperative DES mapping using both ON and AN tasks on the exposed frontal lobe. Intraoperative DES led to repeated errors in the pars opercularis under both tasks, which was spared during resection. Resection was achieved without complications and was extended deep into the white matter reaching the corpus callosum and head of caudate nucleus.

At testing on the third postoperative day, the patient’s performance had slightly, but not significantly worsened in both tasks. MRI scanning confirmed that white matter damage was restricted to the Frontal Aslant Tract in both the ON and the AN network with all other language tracts arching posteriorly away from the resection cavity (**Figures 5E, F**). This suggests that the damage to the Frontal Aslant Tract did not influence naming, and that the majority of the language network remained untouched. The patient was discharged on the fourth postoperative day, with no further cognitive nor physical impairments.

### Clinical Case 6

Case 6 was a 57-year-old male, with a history of having been operated on for an astrocytoma WHO grade II in 2009. Following a biopsy in 2018 with subsequent radio- and chemotherapy, re-resection was now planned. Scans showed contrast-enhanced tumor growth along the previous resection cavity in the middle inferior temporal gyrus. On the preoperative language assessment, he displayed a mild comprehension deficit and moderate impairment in phonological fluency. ON and AN were not impaired at this point. At the preoperative testing day 2 weeks later (one day prior to surgery), his performance had not changed for ON. AN slightly worsened, which although still within normal limits, was now significantly worse than ON (**Table 3** and **Supplementary Material Table S1**).

**Figure 6:**
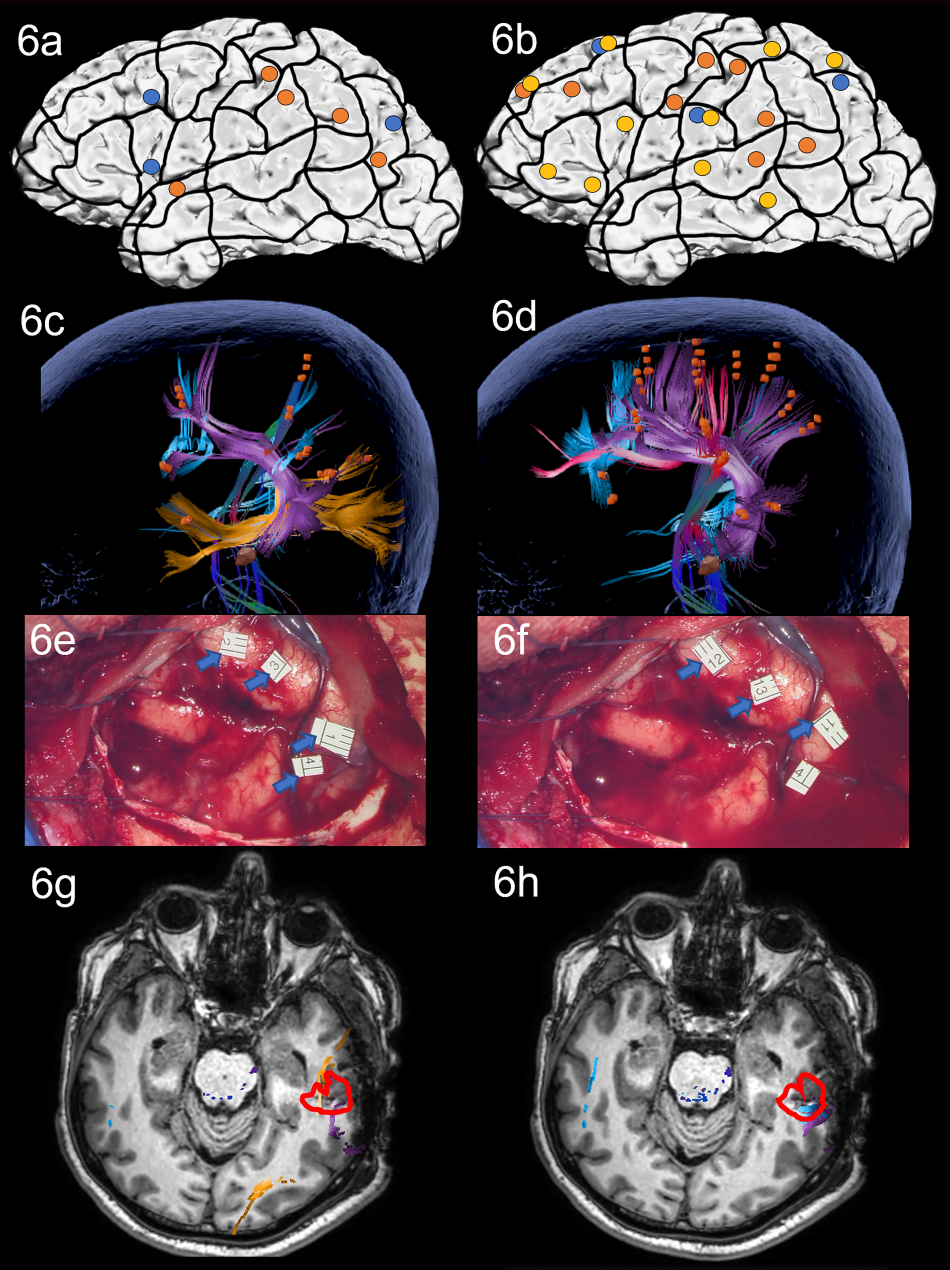
Case 6. Left panels show ON results, right panels AN results. Panels **(A, B)** nTMS stimulation sites that induced errors; no response and hesitations in orange, semantic errors in yellow and performance errors in blue. Panels **(C, D)** Fiber tracking results including the lesion, nTMS cortical endpoints in orange and color-coded tracts with CNT in in direction-coded, here dark blue, AF in purple, SLF in pink, ILF in yellow, and CF in light blue. Panels **(E, F)** Screenshot of intraoperative positive cortical sites with tags 1-3 on the middle and posterior STG and tag 4 on the posterior MTG for ON, and tags 11-13 on the middle and posterior STG for AN. Panels **(G, H)** Overlap of tracking and postoperative T1-weighted imaging after elastic fusion with the resection cavity marked in red.

During nTMS mapping at baseline, an ON network with parietal and frontal areas and one temporal cortical area was revealed, leading to white matter visualization of the AF, ILF, CNT and some parts of the CF (**Figures 6A, C**). AN resulted in many semantic errors and in an overall larger error rate with more frontal and temporal cortical sites and visualization of the AF, SLF, CNT, and CF, but no ventral stream tracts (**Figures 6B, D**).

Due to the spatial relation between the language network and the lesion, the patient was considered for awake surgery using the dual task ON and AN protocol under intraoperative DES. In the exposed middle and posterior temporal area, intraoperative DES led to errors in the STG in both tasks (**Figures 6E, F**: ON errors in tags 1-4; AN errors in tags 11-13), as predicted by nTMS mapping with AN. Moreover, the posterior MTG was found to include language-positive sites for ON (tag 4). Mapping results led to these regions being spared by the surgeon during access to the subcortical lesion. A gross total resection was achieved with no complications.

During testing on the third postoperative day, the patient’s performance was significantly worse in both tasks compared to baseline. ON scores dropped severely to a clinically impaired score and worsened significantly more than AN. AN moderately worsened, with error production correlating with several linguistic values (**Supplementary Material Table S2**). Interestingly, verbs with values for instrumentality (e.g., *the man* ***cuts*** with the instrumental noun *knife*) were particularly affected. Postoperative MRI scans confirmed the overlap of the cavity and the ON network’s AF and ILF and the AN network’s AF and CF (**Figures 6G, H**). This leads to the hypothesis that the harmed tissue in the intersection of the ILF and AF may be responsible for object semantics. Moreover, it could also be an account for the errors in AN for instrumental verbs, as these may be related to object semantics for the instrument.

The patient was discharged with no additional impairments on the third postoperative day.

### Clinical Case 7

Case 7 was a 33-year-old male, admitted to the hospital after having recently suffered from two general epileptic seizures. A lesion was detected in the insular, fronto-basal and temporo-polar regions.

Preoperative baseline language assessment showed no language impairment, as well as normal scores on ON and AN. At the preoperative testing, 3 days later and one day prior to surgery, his performance had not changed significantly (**Table 3** and **Supplementary Material Table S1**).

**Figure 7:**
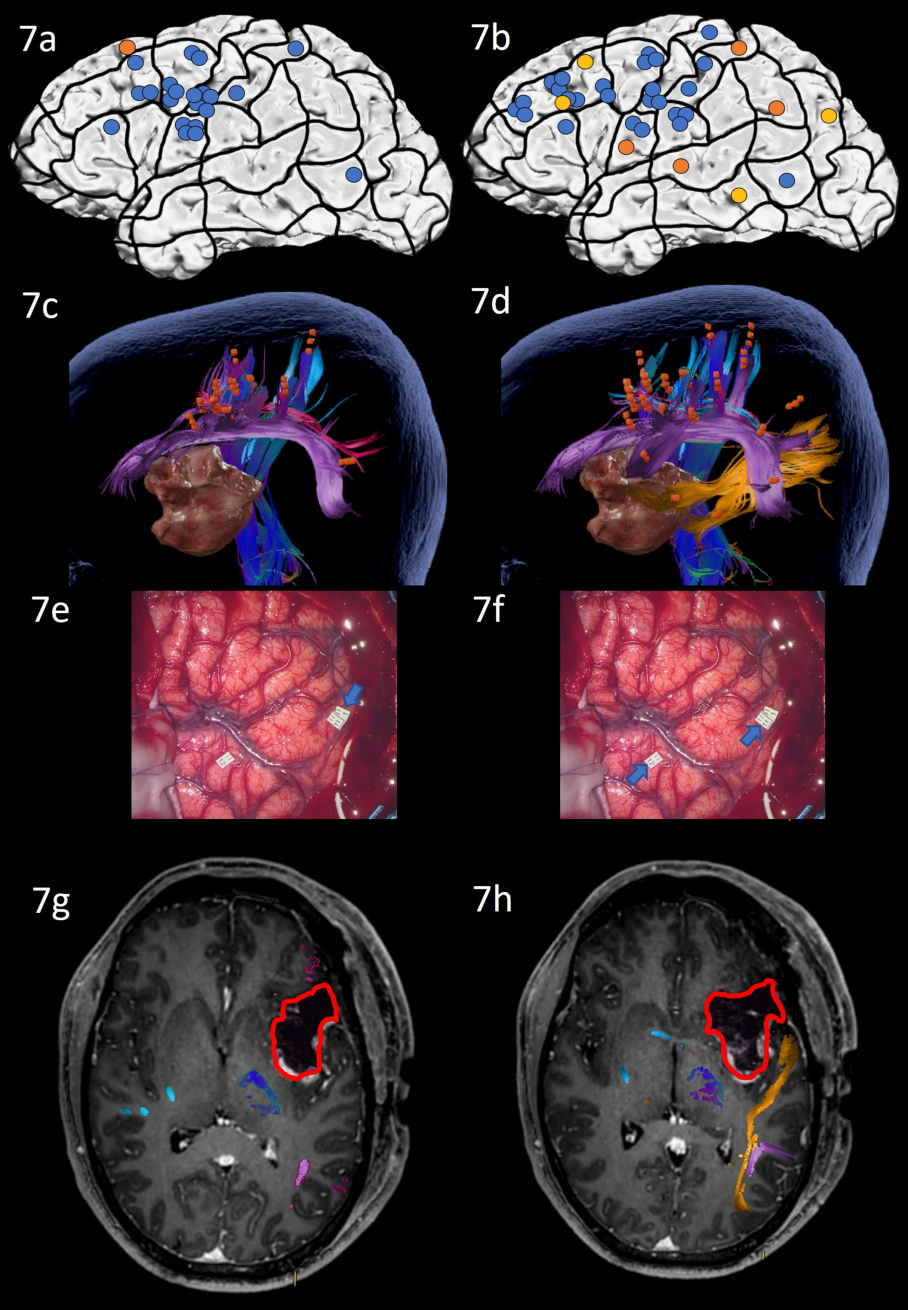
Case 7. Left panels show ON results, right panels AN results. Panels **(A, B)** nTMS stimulation sites that induced errors; no response and hesitations in orange, semantic errors in yellow and performance errors in blue. Panels **(C, D)** Fiber tracking results including the lesion, nTMS cortical endpoints in orange and color-coded tracts with CNT in direction-coded, here dark blue, AF in purple, SLF in pink, ILF in yellow, and CF in light blue. Panels **(E, F)** Screenshot of intraoperative positive cortical sites with tag 1 PoG for ON, and tags 11 + 12 on PoG and the middle STG for AN. Panels **(G, H)** Overlap of tracking and postoperative T1-weighted imaging after elastic with the resection cavity marked in red.

Preoperative nTMS mapping at baseline revealed language-positive cortical sites for ON mainly in the prG and middle frontal gyrus (MFG) resulting in a white matter visualization of the AF, SLF, CNT and CF (**Figures 7A, C**). Apart from these regions, nTMS mapping with AN showed language-positive cortical areas in the parietal and temporal lobe and revealed semantic errors in all 3 lobes, leading to subcortical visualization of the AF, ILF, CNT and CF (**Figures 7B, D**).

Due to the close location of the lesion to the language networks, the surgical procedure included awake intraoperative cortical mapping with DES with ON and AN tasks. The craniotomy exposed inferior frontal and central regions and the anterior superior and middle temporal lobe. Intraoperative DES led to errors in ON in the middle postcentral gyrus (**Figure 7E**: tag 1). AN under DES confirmed this positive site and revealed a second region in the middle STG that repeatedly led to naming errors under stimulation only for AN (**Figure 7F**: tag 11 + 12) and as predicted by nTMS mapping with AN. Subsequent resection avoided these language-positive cortical areas and was extended into the insular lobe.

The patient’s overall condition allowed postoperative testing only on the seventh postoperative day after the operation. His performance on ON had not changed compared to preoperative assessments. AN had significantly worsened and was now clinically impaired, with many semantically incorrect answers (e.g. “*The man … I guess he congratulates somebody*” instead of “*the man claps*” or “*The bird, well, how to say, it flaps its wings*” instead of the target “*The bird flies*”). These errors underlined the patient’s inability to retrieve the target word, while being able to articulate and describe the situation. Postoperative MRI scans showed that the white matter network of ON had been spared entirely and was not in proximity of the cavity, while the AN network’s ILF was in close proximity to the resected tissue (**Figures 7G, H**). The patient was discharged on Day 10, presenting with no further impairments.

## Discussion

Based on the neurolinguistic differences between, and supposed neuroanatomical segregation of, noun and verb knowledge, this series of cases set out to shed light on the benefits of nTMS language mapping from the use of a noun task (ON) and a verb task (AN) in perioperative language delineation in brain tumor patients. We have reported the details of seven cases, each described separately.

While this protocol had been successfully implemented using nTMS in healthy participants (24), the cases reported here show good feasibility of the dual task, ON and AN, protocol in the clinical population at hand: all patients were able to complete the addition of a second mapping with AN, and the extra task added a mere 15 min to the standard workflow of about 45-50 min. The potential added value of the addition of AN to the protocol for pre-, intra-, and postoperative delineation of language is discussed below.

### Preoperative Delineation of the Language State and Network

Due to the higher processing load required during AN compared to ON, as well as AN requiring a larger neuroanatomical circuit (18–21), we hypothesize that these two tasks may be differently affected by tumor growth even before surgical intervention. Moreover, the often milder impairments of glioma patients may not be detectable with ON alone, but only surface after more detailed screening through additional tools that screen, for instance, for more complex retrieval and grammatical skills (Wolthuis et al., subm.). Our study confirms this assumption: while Cases 2, 3, 5 and 7 performed well on ON and AN, as well as the more detailed AAT/ACL screening, the cases displaying a preexisting impairment (Cases 1, 4, and 6, **Table 1**) also performed significantly worse and/or were impaired in AN. This leads to the tentative conclusion that the AN task may be a more sensitive tool to spot the typically more subtle impairments of brain tumor patients. However, we do not suggest the exclusion of ON entirely: although not apparent in our sample, other patients may present with a dissociated deficit of unimpaired verb skills and impaired noun skills [for instance, see cases in (33)].

Regarding the added value of the AN task in preoperative visualization of the language network, the nTMS-only cases (Cases 1-3) show a different pattern to the cases who also underwent intraoperative DES (Cases 4-7). AN results either confirmed the functional cortical and subcortical tissue (Case 1 and 2), already detected during ON, or led to a more comprehensive white matter network with commissural fibers (Case 3). In all three cases, none of the visualized language network components appeared in close proximity to the to-be-resected regions.

In contrast, Cases 4-7 showed a much more divergent network visualization between the two tasks. Firstly, AN showed a higher sensitivity for detecting cortically positive areas in 2 cases (**Table 2**). The sentence context required by this task provides a greater opportunity for language to be disrupted by nTMS (i.e., more lexical items to be retrieved). However, the higher sensitivity of the AN task was still found in healthy participants, when both ON and AN were triggered in sentence context (24). This suggests that rather than an effect of the different context, the difference between ON and AN is that verb retrieval is likely more demanding and more easily disrupted under stimulation of nTMS.

Secondly, the difference in error type pattern between ON and AN further corroborates this: more semantic errors were elicited during AN in three out of our four cases (**Figures 5A, B**, **6A, B**, **7A, B**), originating from a disruption of the lexico-semantic retrieval of verbs. This further justifies the hypothesis that the AN task is lexico-semantically more demanding and is in line with previous findings reporting more semantic errors on the AN task under nTMS in healthy subjects, while errors at the sound level seem to be elicited to a similar degree in ON and AN (16, 17, 24).

In terms of the individual cases, this higher sensitivity for the AN task resulted in more language-positive cortical areas in Case 4, a similar distribution in Case 5, a clear profit from the combination of both tasks with AN for cortical and ON for subcortical visualization in Case 6, and AN was the better candidate for revealing functional cortical and subcortical matter in the proximity of the lesion in Case 7.

In summary, while the ON and AN networks showed high overlap and no close spatial relation to the lesion in Cases 1-3, the lesions of Cases 4-7 appeared rather close to the delineated networks on ON and AN and tumor growth seems to have affected them differently causing segregated reorganization of the two skills. In these cases, mapping with both ON and AN should be considered essential for risk stratification through error count, error quality and resulting white matter analysis after nTMS mapping.

### Overlap With Intraoperative Mapping With DES

Often stressed as the “gold standard” for surgical decision making, DES intraoperative mapping remains the most direct tool for interrogating brain tissue and its functional involvement (34). However, not all patients are eligible for this highly invasive and costly procedure. Moreover, preoperative imaging and mapping aims to reliably predict intraoperative mapping results in order to shorten intraoperative time and to minimize the extent of the exposed cortex even further (4–6). Thus, the question arises how well the nTMS mapping results predicted DES maps in our four cases. The results are suggestive of more potent AN mapping for three cases. In Case 5, both ON and AN nTMS cortical maps failed to predict the negative DES results for most of the frontal lobe. For Case 4, nonetheless, the absence of positive cortical spots on the MFG during DES was in line with AN mapping (**Figures 4A, B**). The DES positive STG and middle MTG in Case 6 overlap with nTMS mapping of AN only (**Figures 6B, E, F**). Finally, in Case 7, AN based nTMS predicted the crucial middle STG region, an area known for verb representation (24, 35–37); this area was not revealed with ON under either DES nor nTMS (**Figures 7A, B, E, F**). The replicability of these results that point towards the higher sensitivity of AN-based nTMS compared ON-based nTMS and their overlap with DES mapping should next be evaluated in further patients with various lesion types and locations.

### Visualization of Postoperative Outcome

The goal of clinical care for tumor patients is a gross total resection in combination with the lowest post-operative deficit. Therefore, our aim in exploring the dual-task ON and AN protocol was to analyze its ability to visualize the preoperative network in relation to the damage to it by the resection: If a network-to-deficit relationship can be captured through nTMS visualization, we may be able to predict and ultimately avoid postoperative language decline in future cases.

In the nTMS-only cases (1-3), this visualization of the functional network and its relation to the postoperative language state were achieved regardless of the task: in each case, the language networks for both ON and AN were spatially distant from the resection cavity. In conformity with this, no new language deficits were found following resection. This finding also underlines how accurately nTMS mapping and subsequent tracking registers the functional circuits of language and the changes to these circuits as a result of plasticity in the context of a tumor: For Cases 2 and 3 in particular, the lesions were located in what would have been thought to be highly eloquent cortical areas which would have rendered a confident and safe tumor removal difficult. However, as nTMS captured the tumor-induced reorganization of the white matter, safe navigation and resection could be performed. These case observations are in agreement with the recent assumption that grey matter loss can be compensated for, while the focus should lie on the preservation of white matter integrity in order to avoid functional damage (38–40).

The added value of mapping with the second task of AN mapping becomes more evident in the remaining cases who additionally underwent intraoperative DES: In Case 4, both ON and AN networks were shown to be in the vicinity of the postoperative cavity (**Figures 4E, F**), particularly the AF and ILF for ON and even more so the ILF for AN. Previous data from DES and nTMS mapping have hinted towards the ventral stream’s involvement in lexico-semantic retrieval (26, 38, 41–51). The presumed more lexico-semantically challenging AN task and network’s close spatial relation to the lesion and cavity in this case are, therefore, in line with the patient’s significant and even more marked drop in performance on AN compared to ON, highlighted further by his struggle with transitive verbs in particular: These verbs, that carry more semantic information about the constituents of the small sentence than intransitive verbs, seemed to be especially troublesome and substantiate the pattern of a lexico-semantic impairment of verbs and the fragility of the ventral stream visualized through AN.

In contrast, in Case 5 all major language white matter tracts showed to be unharmed by the resection, as the surgery could avoid damage to them due to their posteriorly pushed reorganization. Only the Frontal Aslant Tract, known to support fluency in spontaneous speech and not targeted by any of our tasks in particular (52), was depicted in the overlap with the postoperative cavity (**Figures 5E, F**). In accordance with this, no language impairment appeared in Case 5. We conclude that in this case no task was superior to the other in explaining postoperative language function, but that the overall reorganization captured by nTMS-based fiber tracking from both tasks was successful in predicting the postoperative state.

Lastly, Cases 6 and 7 displayed a mirrored pattern in language impairment with a significantly more severe decline in ON for Case 6 and a sole decline in AN for Case 7, while confirming the feasibility of nTMS-based fiber tracking in delineating the network and particularly the vulnerability of the ventral stream ILF. Even though both the AF and CF of the AN network and the AF of the ON network of Case 6 appeared to be damaged by the resection cavity (**Figures 6G, H**), we suspect the possible damage to the ILF of ON may explain the observed language deficit: the substantial drop in ON performance implies a severe deficit of object semantics not only in ON itself, but also affecting verb retrieval of those (instrumental) verbs that utilize objects [**Supplementary Material Table S2**, e.g. *The man* ***cuts*** *(with a knife)*]. This error pattern, that is consistent with a lexico-semantic retrieval deficit, is in accordance with a proposed temporal semantic hub for objects (53, 54). More importantly, however, this finding stresses the benefit of the dual-task protocol for a patient-tailored use of task: firstly, the ability of the method to show the network-to-deficit relationship was proven once more. Secondly, this error pattern of retrieval deficits in both ON and AN could only be substantiated by using the added information from linguistically standardized and marked protocols, that incorporate values such as instrumentality and frequency [for instance: the DULIP, ECCO, VAN-POP (16, 33, 55)].

Case 7, on the other hand, who showed unimpaired postoperative ON and newly impaired AN, is also consistent with the overlap of the network and the postoperative cavity (**Figures 7G, H**): All grey and white matter visualized by ON were unharmed, while, once more, the ILF of the AN network was in close proximity to the resection cavity and likely caused the low score on the AN task.

While our results are consistent with a temporal lobe hub for retrieval of semantics, these two cases stress the feasibility of using nTMS to highlight the vulnerability of the ventral stream on an individual basis: if preoperative visualization predicts proximity of the respective network to the tumor in these areas, special care should be taken of this language skill in order to minimize postoperative deficits even further.

### Future Directions

The observed patterns of impairment of the cases reported here were based on post-operative testing on Day 3. This day often represents one of the first days of a stable cognitive function postoperatively, as well as one of the last days of in-house care before discharge. Even though a feasible and practical choice, this implies that we tested during a transient stage where some of the observed patterns may change or resolve over the next few weeks. However, especially in higher grade gliomas, a new worsening of the language state is also likely to follow due to regrowth of malignant tissue along the resection cavity. Those fragile network regions delineated at this early point in time might once more be affected. Nevertheless, future studies could opt for postoperative testing at the 3-months follow up to target an advanced recovered and possibly re-impaired point in time.

In addition, the implications we can draw at this point are based on a relatively small number of cases with an uneven distribution of lesion sites. Future studies may profit from analysis of larger case series with subgroups clustered by region to be able to make distinct claims of the benefit of each task in a specific region in question.

## Conclusion

As a first instant of using a dual task for nTMS-based language mapping in a peri-operative setting, our case series including the second task of AN delivered promising results. We suggest that the verb task (AN) may be more sensitive in detecting minor pre-existing language impairments preoperatively and in predicting intraoperative positive mapping regions. However, the best grey and white matter visualization of functionally involved tissue may be achieved through implementation of both tasks, since double dissociations across cases are to be expected both behaviorally as well as anatomically.

The method delineated in our paper of nTMS-based mapping and fiber tracking proved capable of capturing network-to-deficit relations, particularly for white matter damage. We recommend that particular care should be dedicated to the preservation of the ventral stream tracts in order to avoid retrieval deficits for both nouns and verbs.

## Data Availability Statement

The raw data supporting the conclusions of this article will be made available by the authors, without undue reservation.

## Ethics Statement

The studies involving human participants were reviewed and approved by the Institutional Review Board of the Technical University of Munich. The patients/participants provided their written informed consent to participate in this study.

## Author Contributions

A-KO, RB, LN, SI, NS, and SK contributed to the conception and design of the study. A-KO, BN, WZ, and NS performed the data collection and analysis. A-KO wrote the manuscript, with contributions and editing by all authors. All authors contributed to the article and approved the submitted version.

## Funding

The study was financed by institutional grants from the Department of Neurosurgery, Klinikum rechts der Isar, School of Medicine, Technische Universität München, Munich, Germany. RB is supported by the Center for Language and Brain NRU Higher School of Economics, RF Government Grant, ag. No. 14.641.31.0004.

## Conflict of Interest

The authors declare that the research was conducted in the absence of any commercial or financial relationships that could be construed as a potential conflict of interest.

## Publisher’s Note

All claims expressed in this article are solely those of the authors and do not necessarily represent those of their affiliated organizations, or those of the publisher, the editors and the reviewers. Any product that may be evaluated in this article, or claim that may be made by its manufacturer, is not guaranteed or endorsed by the publisher.

## References

[1] Lefaucheur JP Picht T. The Value of Preoperative Functional Cortical Mapping Using Navigated TMS. Neurophysiol Clin (2016) 46(2):125–33. doi: 10.1016/j.neucli.2016.05.001 27229765

[2] Ottenhausen M Krieg SM Meyer B Ringel F. Functional Preoperative and Intraoperative Mapping and Monitoring: Increasing Safety and Efficacy in Glioma Surgery. Neurosurg Focus (2015) 38(January):1–13. doi: 10.3171/2014.10.FOCUS14611.Disclosure 25552283

[3] Hannula H Ilmoniemi RJ. Basic Principles of Navigated TMS. In: Navigated Transcranial Magnetic Stimulation in Neurosurgery. Cham: Springer (2017). p. 3–29.

[4] Picht T Krieg SM Sollmann N Rösler J Niraula B Neuvonen T. A Comparison of Language Mapping by Preoperative Navigated Transcranial Magnetic Stimulation and Direct Cortical Stimulation During Awake Surgery. Neurosurgery (2013) 72(5):808–19. doi: 10.1227/NEU.0b013e3182889e01 23385773

[5] Sollmann N Ille S Hauck T Maurer S Negwer C Zimmer C. The Impact of Preoperative Language Mapping by Repetitive Navigated Transcranial Magnetic Stimulation on the Clinical Course of Brain Tumor Patients. BMC Cancer (2015) 15(1):261. doi: 10.1186/s12885-015-1299-5 25885761 PMC4404089

[6] Sollmann N Kelm A Ille S Schröder A Zimmer C Ringel F. Setup Presentation and Clinical Outcome Analysis of Treating Highly Language-Eloquent Gliomas *via* Preoperative Navigated Transcranial Magnetic Stimulation and Tractography. Neurosurg Focus (2018) 44(6):E2. doi: 10.3171/2018.3.FOCUS1838 29852769

[7] Bährend I Muench MR Schneider H Moshourab R Dreyer FR Vajkoczy P. Incidence and Linguistic Quality of Speech Errors: A Comparison of Preoperative Transcranial Magnetic Stimulation and Intraoperative Direct Cortex Stimulation. J Neurosurg (2020) 134(5):1409–18. doi: 10.3171/2020.3.jns193085 32470943

[8] Ille S Sollmann N Hauck T Maurer S Tanigawa N Obermueller T. Combined Noninvasive Language Mapping by Navigated Transcranial Magnetic Stimulation and Functional MRI and Its Comparison With Direct Cortical Stimulation. J Neurosurg (2015) 123(July):1–14. doi: 10.3171/2014.9.JNS14929 25748306

[9] Krieg SM Tarapore PE Picht T Tanigawa N Houde J Sollmann N. Optimal Timing of Pulse Onset for Language Mapping With Navigated Repetitive Transcranial Magnetic Stimulation. NeuroImage (2014) 100:219–36. doi: 10.1016/j.neuroimage.2014.06.016 24945663

[10] Takahashi S Vajkoczy P Picht T. Navigated Transcranial Magnetic Stimulation for Mapping the Motor Cortex in Patients With Rolandic Brain Tumors. Neurosurg Focus (2013) 34(4):1–7. doi: 10.3171/2013.1.FOCUS133 23544409

[11] Tarapore PE Findlay AM Honma SM Mizuiri D Houde JF Berger MS. Language Mapping With Navigated Repetitive TMS: Proof of Technique and Validation. NeuroImage (2013) 82:260–72. doi: 10.1016/j.neuroimage.2013.05.018 PMC3759608 23702420

[12] Negwer C Ille S Hauck T Sollmann N Maurer S Kirschke JS. Visualization of Subcortical Language Pathways by Diffusion Tensor Imaging Fiber Tracking Based on rTMS Language Mapping. Brain Imaging Behav (2017) 11(3):899–914. doi: 10.1007/s11682-016-9563-0 27323766

[13] Raffa G Conti A Scibilia A Sindorio C Quattropani M Visocchi M. Functional Reconstruction of Motor and Language Pathways Based on Navigated Transcranial Magnetic Stimulation and DTI Fiber Tracking for the Preoperative Planning of Low Grade Glioma Surgery: A New Tool for Preservation and Restoration of Eloquent Network. in. Trends Reconstr Neurosurg - Neurorehabil Restor Reconstruction (2017) 124:251–62. doi: 10.1007/978-3-319-39546-3_37 28120081

[14] Sollmann N Negwer C Ille S Maurer S Hauck T Kirschke JS. Feasibility of nTMS-Based DTI Fiber Tracking of Language Pathways in Neurosurgical Patients Using a Fractional Anisotropy Threshold. J Neurosci Methods (2016) 267:45–54. doi: 10.1016/j.jneumeth.2016.04.002 27059128

[15] Sollmann N Meyer B Krieg SM. Implementing Functional Preoperative Mapping in the Clinical Routine of a Neurosurgical Department: Technical Note. World Neurosurg (2017) 103:94–105. doi: 10.1016/j.wneu.2017.03.114 28377253

[16] Ohlerth A-K Valentin A Vergani F Ashkan K Bastiaanse R. The Verb and Noun Test for Peri-Operative Testing (VAN-POP): Standardized Language Tests for Navigated Transcranial Magnetic Stimulation and Direct Electrical Stimulation. Acta Neurochir (2020) 162(2):397–406. doi: 10.1007/s00701-019-04159-x 31823119 PMC6982630

[17] Rofes A Miceli G. Language Mapping With Verbs and Sentences in Awake Surgery: A Review. Neuropsychol Rev (2014) 24(2):185–99. doi: 10.1007/s11065-014-9258-5 24736866

[18] Bastiaanse R Wieling M Wolthuis N. The Role of Frequency in the Retrieval of Nouns and Verbs in Aphasia. Aphasiology (2016) 30(11):1221–39. doi: 10.1080/02687038.2015.1100709

[19] Bastiaanse R Van Zonneveld R. Broca’s Aphasia, Verbs and the Mental Lexicon. Brain Lang (2004) 90(1–3):198–202. doi: 10.1016/S0093-934X(03)00432-2 15172537

[20] Crepaldi D Berlingeri M Cattinelli I Borghese NA Luzzatti C Paulesu E. Clustering the Lexicon in the Brain: A Meta-Analysis of the Neurofunctional Evidence on Noun and Verb Processing. Front Hum Neurosci (2013) 7:303(JUN). doi: 10.3389/fnhum.2013.00303 23825451 PMC3695563

[21] Vigliocco G Vinson DP Druks J Barber H Cappa SF. Nouns and Verbs in the Brain: A Review of Behavioural, Electrophysiological, Neuropsychological and Imaging Studies. Neurosci Biobehav Rev (2011) 35:407–26. doi: 10.1016/j.neubiorev.2010.04.007 20451552

[22] Havas V Gabarrós A Juncadella M Rifa-Ros X Plans G Acebes JJ. Electrical Stimulation Mapping of Nouns and Verbs in Broca’s Area. Brain Lang (2015) 145:53–63. doi: 10.1016/j.bandl.2015.04.005 25957505

[23] Lubrano V Filleron T Démonet J-F Roux F-E. Anatomical Correlates for Category-Specific Naming of Objects and Actions: A Brain Stimulation Mapping Study. Hum Brain Mapp (2014) 35:429–43. doi: 10.1002/hbm.22189 PMC6869226 23015527

[24] Ohlerth A-K Bastiaanse R Negwer C Sollmann N Schramm S Schröder A. Bihemispheric Navigated Transcranial Magnetic Stimulation Mapping for Action Naming Compared to Object Naming in Sentence Context. Submitted Publication (2021) 11(9):1190. doi: 10.3390/brainsci11091190 PMC8469437 34573211

[25] Rofes A Spena G Talacchi A Santini B Miozzo A Miceli G. Mapping Nouns and Finite Verbs in Left Hemisphere Tumors: A Direct Electrical Stimulation Study Mapping Nouns and Finite Verbs in Left Hemisphere Tumors: A Direct Electrical Stimulation Study International Doctorate in Experimental Approaches to Language (2017). Available at: 10.1080/13554794.2017.1307418. 28347212

[26] Ohlerth AK Bastiaanse R Negwer C Sollmann N Schramm S Schröder A. Benefit of Action Naming Over Object Naming for Visualization of Subcortical Language Pathways in Navigated Transcranial Magnetic Stimulation-Based Diffusion Tensor Imaging-Fiber Tracking. Front Hum Neurosci (2021) 15:748274. doi: 10.3389/fnhum.2021.748274 34803634 PMC8603927

[27] Krieg SM Lioumis P Mäkelä JP Wilenius J Karhu J Hannula H. Protocol for Motor and Language Mapping by Navigated TMS in Patients and Healthy Volunteers; Workshop Report. Acta Neurochir (2017) 159(7):1187–95. doi: 10.1007/s00701-017-3187-z 28456870

[28] Ojemann G Ojemann J Lettich E Berger M. Cortical Language Localization in Left, Dominant Hemisphere. An Electrical Stimulation Mapping Investigation in 117 Patients. 1989. J Neurosurg (1989) 71(2):316–26. doi: 10.3171/JNS/2008/108/2/0411 2769383

[29] Picht T Kombos T Gramm HJ Brock M Suess O. Multimodal Protocol for Awake Craniotomy in Language Cortex Tumour Surgery. Acta Neurochir (2006) 148(2):127–37. doi: 10.1007/s00701-005-0706-0 16374563

[30] Ille S Schwendner M Zhang W Schroeder A Meyer B Krieg SM. Tractography for Subcortical Resection of Gliomas Is Highly Accurate for Motor and Language Function: Iomri-Based Elastic Fusion Disproves the Severity of Brain Shift. Cancers (2021) 13(8):1787. doi: 10.3390/cancers13081787 33918598 PMC8068819

[31] Sollmann N Fuss-Ruppenthal S Zimmer C Meyer B Krieg SM. Investigating Stimulation Protocols for Language Mapping by Repetitive Navigated Transcranial Magnetic Stimulation. Front Behav Neurosci (2018) 12:197. doi: 10.3389/fnbeh.2018.00197 30250427 PMC6139335

[32] Crawford JR Garthwaite PH Porter S. Point and Interval Estimates of Effect Sizes for the Case-Controls Design in Neuropsychology: Rationale, Methods, Implementations, and Proposed Reporting Standards. Cogn Neuropsychol (2010) 27(3):245–60. doi: 10.1080/02643294.2010.513967 20936548

[33] De Witte E Satoer D Robert E Colle H Verheyen S Visch-Brink E. The Dutch Linguistic Intraoperative Protocol: A Valid Linguistic Approach to Awake Brain Surgery. Brain Lang (2015) 140:35–48. doi: 10.1016/j.bandl.2014.10.011 25526520

[34] De Witt Hamer PC Robles SG Zwinderman AH Duffau H Berger MS. Impact of Intraoperative Stimulation Brain Mapping on Glioma Surgery Outcome: A Meta-Analysis. J Clin Oncol (2012) 30(20):2559–65. doi: 10.1200/JCO.2011.38.4818 22529254

[35] Corina DP Gibson EK Martin R Poliakov A Brinkley J Ojemann GA. Dissociation of Action and Object Naming: Evidence From Cortical Stimulation Mapping. Hum Brain Mapp (2005) 24(1):1–10. doi: 10.1002/hbm.20063 15593268 PMC6871733

[36] Hauck T Tanigawa N Probst M Wohlschlaeger A Ille S Sollmann N. Task Type Affects Location of Language- Positive Cortical Regions by Repetitive Navigated Transcranial Magnetic Stimulation Mapping. PloS One (2015) 10(4):1–21. doi: 10.1371/journal PMC4415771 25928744

[37] Hernandez-Pavon JC Mäkelä N Lehtinen H Lioumis P Mäkelä JP. Effects of Navigated TMS on Object and Action Naming. Front Hum Neurosci (2014) 8:660(660). doi: 10.3389/fnhum.2014.00660 25228868 PMC4151040

[38] Bello L Gallucci M Fava M Carrabba G Giussani C Acerbi F. Intraoperative Subcortical Language Tract Mapping Guides Surgical Removal of Gliomas Involving Speech Areas. Neurosurgery (2007) 60(1):67–80. doi: 10.1227/01.NEU.0000249206.58601.DE 17228254

[39] Duffau H. The Huge Plastic Potential of Adult Brain and the Role of Connectomics: New Insights Provided by Serial Mappings in Glioma Surgery. Cortex (2014) 58:325–37. doi: 10.1016/J.CORTEX.2013.08.005 24050218

[40] Trinh VT Fahim DK Shah K Tummala S McCutcheon IE Sawaya R. Subcortical Injury Is an Independent Predictor of Worsening Neurological Deficits Following Awake Craniotomy Procedures. Neurosurgery (2013) 72(2):160–9. doi: 10.1227/NEU.0b013e31827b9a11 23147778

[41] Friederici AD. The Cortical Language Circuit: From Auditory Perception to Sentence Comprehension. Trends Cogn Sci (2012) 16(5):262–8. doi: 10.1016/j.tics.2012.04.001 22516238

[42] Friederici AD. White-Matter Pathways for Speech and Language Processing. Vol 129. 1st ed. Elsevier B.V (2015). doi: 10.1016/B978-0-444-62630-1.00010-X 25726269

[43] Hickok G Poeppel D. Dorsal and Ventral Streams: A Framework for Understanding Aspects of the Functional Anatomy of Language. Cognition (2004) 92(1–2):67–99. doi: 10.1016/j.cognition.2003.10.011 15037127

[44] Hickok G Poeppel D. The Cortical Organization of Speech Processing. Nat Rev Neurosci (2007) 8(5):393–402. doi: 10.1038/nrn2113 17431404

[45] Ueno T Saito S Rogers TT Ralph MA. Lichtheim 2: Synthesizing Aphasia and the Neural Basis of Language in a Neurocomputational Model of the Dual Dorsal-Ventral Language Pathways. Neuron (2011) 72(2):385–96. doi: 10.1016/j.neuron.2011.09.013 22017995

[46] Bello L Gambini A Castellano A Carrabba G Acerbi F Fava E. Motor and Language DTI Fiber Tracking Combined With Intraoperative Subcortical Mapping for Surgical Removal of Gliomas. Neuroimage (2008) 39(1):369–82. doi: 10.1016/j.neuroimage.2007.08.031 17911032

[47] Bello L Castellano A Fava E Casaceli G Riva M Scotti G. Intraoperative Use of Diffusion Tensor Imaging Fiber Tractography and Subcortical Mapping for Resection of Gliomas: Technical Considerations. Neurosurg Focus (2010) 28(2):1–14. doi: 10.3171/2009.12.FOCUS09240 20121441

[48] De Witt Hamer PC Moritz-Gasser S Gatignol P Duffau H. Is the Human Left Middle Longitudinal Fascicle Essential for Language? A Brain Electrostimulation Study. Hum Brain Mapp (2011) 32(6):962–73. doi: 10.1002/hbm.21082 PMC6870476 20578169

[49] Duffau H Gatignol P Mandonnet E Peruzzi P Tzourio-Mazoyer N Capelle L. New Insights Into the Anatomo-Functional Connectivity of the Semantic System: A Study Using Cortico-Subcortical Electrostimulations. Brain (2005) 128(4):797–810. doi: 10.1093/brain/awh423 15705610

[50] Leclercq D Duffau H Delmaire C Capelle L Gatignol P Ducros M. Comparison of Diffusion Tensor Imaging Tractography of Language Tracts and Intraoperative Subcortical Stimulations: Clinical article. J Neurosurg (2010) 112(3):503–11. doi: 10.3171/2009.8.JNS09558 19747052

[51] Maldonado IL Moritz-Gasser S de Champfleur NM Bertram L Moulinié G Duffau H. Surgery for Gliomas Involving the Left Inferior Parietal Lobule: New Insights Into the Functional Anatomy Provided by Stimulation Mapping in Awake Patients: Clinical article. J Neurosurg (2011) 115(4):770–9. doi: 10.3171/2011.5.JNS112 21699481

[52] Dick AS Garic D Graziano P Tremblay P. The Frontal Aslant Tract (FAT) and Its Role in Speech, Language and Executive Function. Frontal Aslant Tract (FAT) Its Role Speech Lang Exec Funct (2018) 111:148–63. doi: 10.1101/249912 PMC6461388 30481666

[53] Indefrey P. The Spatial and Temporal Signatures of Word Production Components: A Critical Update. Front Psychol (2011) 2:1–16. doi: 10.1016/j.cognition.2002.06.001 22016740 PMC3191502

[54] Indefrey P Levelt WJM. The Neural Correlates of Language Production. New Cogn Neurosci (2000) 2000:845–65.

[55] Rofes A de Aguiar V Miceli G. A Minimal Standardization Setting for Language Mapping Tests: An Italian Example. Neuro Sci (2015) 36(7):1113–9. doi: 10.1007/s10072-015-2192-3 25851729

