# Supplementary material for: Dual-Task nTMS Mapping to Visualize the Cortico-Subcortical Language Network and Capture Postoperative Outcome—A Patient Series in Neurosurgery
Source: Front Oncol. 2022 Jan 21;11:788122. doi: 10.3389/fonc.2021.788122 (PMC8814635; doi:10.3389/fonc.2021.788122)
Supplement: Supplementary file 1 [file Table_1.pdf]

Supplementary Material

*Table S1: Proportion of correctly and fluently named items in ON and AN across testing points, as reported in Table 2 of the main document with more information for between test comparison: Scores are reported in proportions of correctly named items during ON and AN. Scores that indicate a clinical impairment compared to healthy normative data (Singlims, Crawford et al., 2010) are marked by a hashtag (#). Healthy controls performed at 0.884 ± 0.046 (range 0.8-0.933) for ON (*Sollmann, Fuss-Ruppenthal, et al., 2018*) and at 0.852 ± 0.059 (range 0.667-0.933) for AN (Ohlerth et al., 2021). Significant difference between tasks (Revised Standardized Difference Test (RSDT), Crawford et al., 2010) is marked by an asterisk (*). Case 1-3 were not operated awake and, hence, the tests were not administered (N/A) 1 Day preoperatively.*

Case	Test	Baseline	Impairment compared to healthy controls at baseline	Difference between ON and AN at baseline	1 Day Pre-operative	Impairment compared to healthy controls pre-operative	Difference between ON and AN preop	3 Days Post-operative	Impairment compared to healthy controls postop	Difference between ON and AN post-operative	Sig. Difference between Baseline and Preoperative	Sig. Difference between Baseline and Postoperative	Difference in Decline between ON and AN (Unpaired MWU)
1	ON	**0.813***	0.149	**0.018***	N/A	N/A	N/A	**0.838***	0.341	**0.016***	N/A	0.790	0.967
	AN	**0.573*#**	**<0.001***		N/A	N/A		**0.600*#**	**0.001***		N/A	0.831	
2	ON	0.850	0.485	0.401	N/A	N/A	N/A	0.825	0.230	0.262	N/A	0.803	0.702
	AN	0.747	0.097		N/A	N/A		0.693#	**0.017**		N/A	0.522	
3	ON	0.975	0.069	0.243	N/A	N/A	N/A	0.988	0.041	0.125	N/A	0.999	0.567
	AN	0.880	0.648		N/A	N/A		0.867	0.811		N/A	0.999	
4	ON	0.925	0.395	0.088	**0.868***	0.999		**0.747***	0.056	**0.001**	0.147*	**0.037**	**0.005***
	AN	0.773	0.208		**0.653*#**	**0.004**	**<0.001**	**0.427*#**	**<0.001**		**0.039***	**<0.001**	
5	ON	**0.963***	0.113	**0.008***	**0.934***	0.999	**<0.001**	0.888	0.938	0.053	0.505	0.114	0.555
	AN	**0.733***	0.064		**0.680*#**	**0.010**		0.707#	**0.026**		0.480	0.831	
6	ON	0.888	0.938	0.558	**0.945***	0.999	**<0.001**	**0.350*#**	**<0.001**	**<0.001**	0.263	**<0.001**	**0.004***
	AN	0.813	0.530		**0.733***	0.064		**0.533*#**	**<0.001**		0.286	**<0.001**	
7	ON	0.913	0.551	0.178	**0.967***	0.999	**<0.001**	**0.875***	0.886	**0.003**	0.192	0.606	**0.040***
	AN	0.787	0.293		**0.827***	0.680		**0.587*#**	**0.0001**		0.580	**0.009**	

Table S2: Bonferroni corrections of correlations between test scores and linguistic values of items.

Case	Test	Linguistic value	Spearman correlation coefficient	p-value after Bonferroni correction
4	Postop AN	Transitivity	-0.3915824	0.004
6	Postop ON	Frequency	0.4142422	0.001
	Postop AN	Age of Acquisition	-0.3101887	0.047
	Postop AN	Instrumentality	-0.3392857	0.020
